# Tunable electron transfer rate in a CdSe/ZnS-based complex with different anthraquinone chloride substitutes

**DOI:** 10.1038/s41598-019-44325-w

**Published:** 2019-05-23

**Authors:** Huifang Zhao, Chaofan Sun, Hang Yin, Yuanzuo Li, Jianbo Gao, Ying Shi, Mengtao Sun

**Affiliations:** 1 Institute of Atomic and Molecular Physics, Jilin University, Changchun, 130012 China; 2 College of Science, Northeast Forestry University, Harbin, 150040 Heilongjiang China; 3 Ultrafast Photophysics of Quantum Devices Laboratory, Department of Physics and Astronomy, Clemson University, South Carolina, 29634 USA; 4 School of Mathematics and Physics, Center for Green Innovation, Beijing Key Laboratory for Magneto-Photoelectrical Composite and Interface Science, University of Science and Technology Beijng, Beijing, 100083 China

**Keywords:** Nanophotonics and plasmonics, Ultrafast photonics, Organic-inorganic nanostructures, Atomic and molecular interactions with photons

## Abstract

We use femtosecond transient absorption spectroscopy to study ultrafast electron transfer (ET) dynamics in a model donor and acceptor system using CdSe/ZnS core/shell structure quantum dots (QDs) as donors and anthraquinone (AQ) molecules as acceptors. The ET rate can be enhanced by decreasing the number of chlorine substituents in the AQ molecules because that increases the driving force, which is the energy level offset between the conduction band energy of CdSe/ZnS and the lowest upper molecular orbital potential of AQ derivatives, as confirmed by cyclic voltammetry measurements. However, the electronic coupling between the QDs and AQ derivatives, and the sum of reorganization energy of AQ molecules and solvent calculated by density functional theory are not the main reasons for the change in ET rate in three systems. Our findings provide new insights into selecting an acceptor molecule and will be useful in tuning ET processes for advanced QD-based applications.

## Introduction

The electron transfer (ET) process is one of the most fundamental mechanisms in quantum dots (QDs), which are used for a wide range of nanotechnology applications such as bioimaging^1–4^, lasing^5–7^, light-emitting diodes^8–10^, molecular device operation^11–13^, and solar cells^14–17^. The ET process from photoexcited QDs to external acceptors through an interface^18,19^ is one of the primary mechanisms associated with the functionality and efficiency of QD devices. Therefore, it is important to understand the ET process, to evaluate its dependence on key factors, and promote it in a QD-based system to further advance QD-based applications. For instance, Lian’s group tuned the shell thickness in the CdSe/ZnS core/shell structure to slow down the ET rate by changing the radial distribution of the electron and hole wavefunctions^12^. Wise’s group has adjusted various solvents in PbS-10-dodecylanthracence-9-thiol (DAT) system to dramatically increase the ET rate by tuning the solvent dielectric constant^20^. Different QDs tuned by wavefunction engineering can be used to control exciton dynamics and ET properties^21,22^. By changing molecular acceptors, Alivisatos *et al*. successfully tuned the hole transfer rate^23^. For the ET process, most groups have focused on changing the QDs and solvents. However, the role of substituents in acceptor molecules is unknown.

In this report, we studied anthraquinone (AQ) molecules with chlorine substituents. Three AQ derivatives with chlorine substituents at positions 1,4,5,8 and 1,8, and 1, namely 1,4,5,8-tetrachloroanthraquinone (1,4,5,8-TTAQ), 1,8-dichloroanthraquinone (1,8-DCAQ), and 1-chloroanthraquinone (1-CAQ) respectively, were introduced as electron acceptors, and CdSe/ZnS core/shell QDs were introduced as electron donors. Ultrafast transient absorption (TA) spectroscopy, a robust tool for tracking real-time charge transfer dynamics in QD-molecular systems, was used to examine the ET process in these QD-molecular complexes. We found that the ET rates in the QD-1,4,5,8-TTAQ, QD-1,8-DCAQ, and QD-1-CAQ complexes exhibit a clear increase as the number of chloride substituents decreased. The framework of Marcus theory and density functional theory (DFT) calculations indicated that the electronic coupling between the QDs and AQ derivatives, and the sum of the reorganization energy of the AQ molecules and solvent are not the main reasons for the change in the ET rate in the three systems. The increasing ET rate is due to the enhanced driving force resulting from the energy mismatch between the QDs and acceptor molecules, as confirmed by cyclic voltammetry (CV) measurements. Such important findings can potentially be applied for further design of QD-based materials.

## Results and Discussion

**Figure 1:**
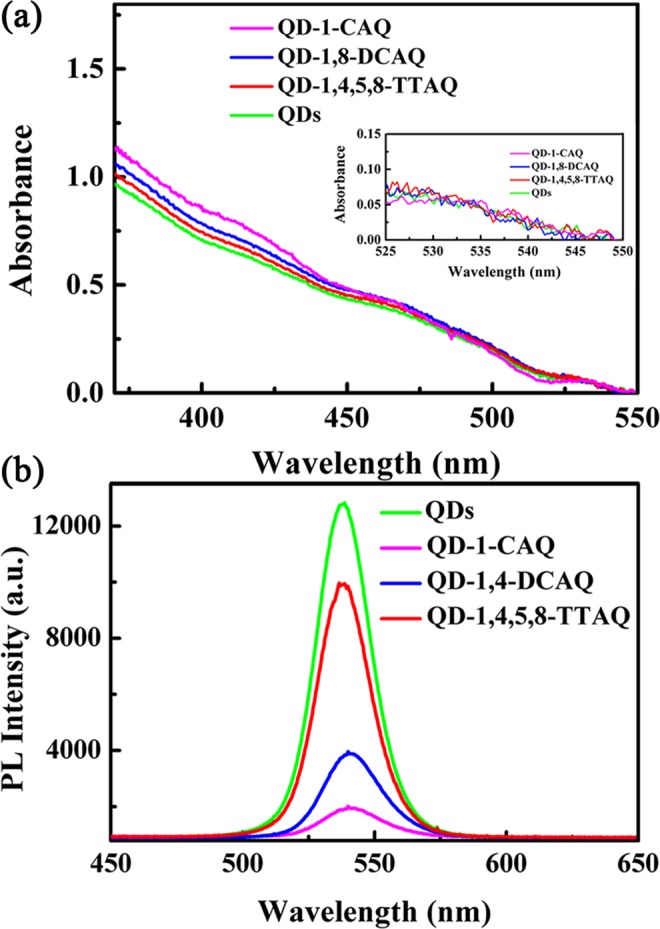
(**a**) Steady-state absorption and (**b**) photoluminescence (PL) spectra of the QDs and different QD-AQ complexes in CHX.

The steady-state absorption and photoluminescence (PL) spectra of QDs and different QD-AQ complexes in cyclohexane (CHX) are shown in Fig. 1. (QD characterization can be found in Supplementary Information Fig. S1). The spectra, as demonstrated in Fig. 1(a), present a broad absorption from only the QD solution (green), which is consistent with a previous study^12^. The same broad absorption is essentially unchanged for all QD-AQ complexes. As shown in Fig. 1 insert, the integrated areas of the first exciton absorption band corresponding to the absorbance at approximately 533 nm are the same for all three systems, which indicates the samples have the same QD concentration^12^. The absorption of the AQ derivatives is shown in Fig. S2, and the results indicate that a 400 nm wavelength laser can only excite the QDs, not the AQ derivative molecules. The fluorescence peaks of the QDs and QD-AQ complexes are all centred at 538 nm as shown in Fig. 1(b). However, while the fluorescence peak position remains constant, the fluorescence intensities are dramatically quenched by different degrees. In particular, the degree of quenching for QD-1,4,5,8-TTAQ, QD-1,8-DCAQ, and QD-1-CAQ reached up to 24.07%, 74.95% and 90.88% respectively, relative to the fluorescence intensity of the pure QDs. Therefore, the PL quenching indicates that the ET from the QDs to the AQ derivatives, and the ET process in the QD-1-CAQ complex would be the strongest^20,24,25^.

**Figure 2:**
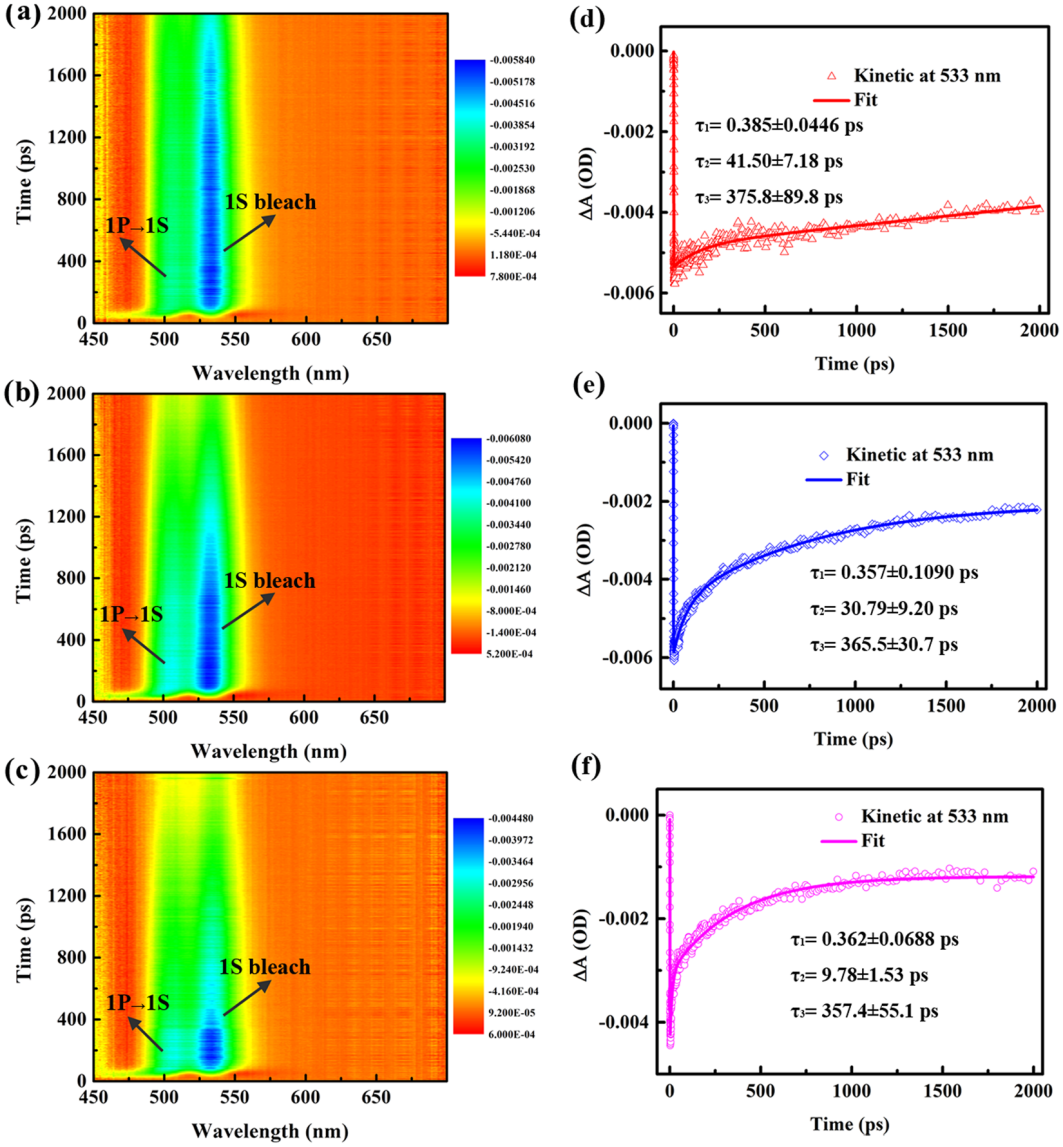
2D image plots of the TA spectra for QD-1,4,5,8-TTAQ (**a**), QD-1,8-DCAQ (**b**), and QD-1-CAQ (**c**) in CHX under 400 nm wavelength pump laser excitation. The TA kinetics of QD-1,4,5,8-TTAQ (**d**), QD-1,8-DCAQ (**e**), and QD-1-CAQ (**f**) with a 533 nm wavelength probe laser.

To gain a deeper understanding of the photoexcited ET dynamics in QD-AQ complexes, we used femtosecond TA spectra to study the ultrafast charge dynamics. A femtosecond ultraviolet pump/white-light continuum probe scheme is employed, and the details of the pump-probe experiments are given in the experimental setup. Figure 2(a–c) show the 2 dimensional (2D) image plot of transient absorption for QD-AQ complexes from 0 to 2 ns in the wavelength region of 450–650 nm. The 2D image plot as a function of wavelength and time delay shows a global overview of the excited dynamics after photoexcitation. There are two peaks centred at 533 nm and 505 nm in the three TA spectra, which have been attributed to the bleaches of the first exciton (1S_e_) and 1P_e_ exciton, respectively^12,26^. More remarkably, a clear decrease in the 1S_e_ bleach signal intensity in QD-1,4,5,8-TTAQ, QD-1,8-DCAQ, and QD-1-CAQ can be observed. This result is in good agreement with the fluorescence quenching result, as indicated by the steady-state PL spectra in Fig. 1(b).

The TA spectra of QD-AQ complexes at different delay times are depicted in Fig. S3 of the Supplementary Information. The 1S_e_ bleach signal amplitude showed an obvious decrease as the delay time increased from 5 ps to 2 ns. In addition to the amplitude changes, the peak of the 1S_e_ bleach band redshifted to different degrees with increasing delay times between 5 ps to 2 ns. The redshift of QD-1-CAQ is more obvious than that of QD-1,8-DCAQ and QD-1,4,5,8-TTAQ. This redshift might be due to a reduction in the population of excited QDs and an increase in the population of the charge transfer states which are both induced by the ET from excited QDs to AQ derivatives^12^. We found that the lifetime of 1S electrons in QDs gradually decreased from QD-1,4,5,8-TTAQ, QD 1,8-DCAQ to QD 1-CAQ, which is consistent with the decrease in the 1S_e_ bleach signal intensity and steady-state PL intensity.

The lifetime of the dynamics processes of QD-AQ complexes can be extracted by a typical dynamics fitting, as shown in Fig. 2(d–f), and the insets are the fitting parameters. Since the 450–700 nm wavelength white-light continuum probe yields essentially the same TA kinetics for each sample, we show here a set of representative data taken at the peak wavelength of 533 nm^27^. The kinetic curves for the three QD-AQ complexes are well fitted by three exponentials with lifetimes of τ_1_ in the hundreds-of-femtoseconds range, τ_2_ in the tens-of-picoseconds range and τ_3_ in the hundreds-of-picoseconds range. The fast component τ_1_ can be attributed to the cooling time of electrons from 1P_e_ to 1S_e_^12,26,28^, τ_2_ can be attributed to the lifetime of the ET process^12^, and the slow component τ_3_ can be attributed to the lifetime of Auger recombination (AR)^29–31^. The fitting results of the dynamics demonstrate that the ET process becomes slower with the addition of chlorine substituents, *i.e*., 1-CAQ >1,8-DCAQ >1,4,5,8-TTAQ. To address the transfer dynamics, we use the frame work of Marcus ET theory to understand the above trend.

In a QD-molecular donor-accepter system, we can assume that a hole remains unchanged when an electron is transferred to the accepter reported^12,32^ The process can be described by Marcus ET theory^33–35^, and the ET rate is given by

1 $${{k}}_{{ET}}=\frac{4{\pi }^{2}}{{h}}{({{H}}_{{DA}})}^{2}\frac{1}{{(4\pi {\lambda }{{k}}_{{B}}{T})}^{1/2}}{\exp }(\,-\,\frac{{({\lambda }+{\rm{\Delta }}{{G}}^{0})}^{2}}{4{\lambda }{{k}}_{{B}}{T}})$$

where H_DA_ is the electronic coupling between the initial and final state of the donor-acceptor system, λ is the reorganization energy (RE), ΔG° is the driving force, k_B_ is the Boltzmann constant and T is the temperature. Three factors H_DA,_ RE, and ΔG° will be introduced for every QD-AQ complex to discuss the influence of them on ET rate.

The RE consists of two parts and can be expressed in detail as λ = λ_i_ + λ_0_, where λ_i_ is the RE of the acceptor molecular and λ_0_ is the RE of the solvent because the contribution from the QDs is negligible^36^. The acceptor molecular RE can be expressed by the following equation^37,38^:

2 $${{\rm{\lambda }}}_{{\rm{i}}}=({{\rm{E}}}_{0}^{-}-{{\rm{E}}}_{-})+({{\rm{E}}}_{-}^{0}-{{\rm{E}}}_{0})$$

where ${{\rm{E}}}_{0}^{-}$ is the energy of the anion calculated using the optimized structure of the neutral molecule; ${{\rm{E}}}_{-}$ is the energy of the anion calculated using the optimized anion structure; ${{\rm{E}}}_{-}^{0}$ is the energy of the neutral molecule calculated in the anionic state; ${{\rm{E}}}_{0}$ is the energy of the neutral molecule in the ground state. The anionic and neutral molecular energies of 1,4,5,8-TTAQ, 1,8-DCAQ and 1-CAQ were calculated at the B3LYP/6–311 G level and the reorganization energies of the electrons were obtained by using the above equation. For the solvent RE (λ_0_) can be estimated using the dielectric continuum model by the following equation^34,39^:

3 $${{\rm{\lambda }}}_{0}=\frac{{{\rm{e}}}^{2}}{4{{\rm{\pi }}{\rm{\varepsilon }}}_{0}}(\frac{1}{{{\rm{\varepsilon }}}_{{\rm{op}}}}-\frac{1}{{{\rm{\varepsilon }}}_{{\rm{s}}}})(\frac{1}{{{\rm{d}}}_{{\rm{D}}}}+\frac{1}{{{\rm{d}}}_{{\rm{A}}}}-\frac{1}{{{\rm{r}}}_{{\rm{DA}}}})$$

where ε_0_ is the dielectric constant under vacuum; ε_op_ and ε_s_ are the optical and static dielectric constants of the solvent, respectively; d_D_ and d_A_ are the diameters of the spherical donor and acceptor cavities, respectively; and r_DA_ is the centre-to-centre distance between the donor and acceptor. The radius of the AQ derivatives is shown in Table 1. We calculated the RE of the three QD-AQ (λ_i_) complexes and the solvent (λ_0_) as tabulated in Table 2. The sum RE (λ) of the acceptor molecular (λ_i_) and the solvent (λ_0_) in QD-1,4,5,8-TTAQ, QD-1,8-DCAQ, and QD-1-CAQ, indicates that there are very small differences in the sum RE (λ) of the three QD-AQ derivative systems. Thus, we can conclude that the RE of the solvent and acceptor molecule is not the main reason for the change in the ET time caused by the addition of chlorine.

**Table 1:** Volumes of 1,4,5,8-TTAQ, 1,8-DCAQ, and 1-CAQ.

System	Mole volume (cm^3^/mol)	Radius (nm)
1,4,5,8-TTAQ	199.604	0.428
1,8-DCAQ	170.978	0.408
1-CAQ	161.890	0.400

**Table 2:** RE of QD-1,4,5,8-TTAQ, QD-1,8-DCAQ, and QD-1-CAQ and the solvent CHX in the three AQ derivatives systems.

System	λ_i_ (eV)	λ_0_ (eV)	λ (eV)
QD-1,4,5,8-TTAQ	0.350	0.0438	0.3938
QD-1,8-DCAQ	0.356	0.0461	0.4021
QD-1-CAQ	0.359	0.0468	0.4058

**Figure 3:**
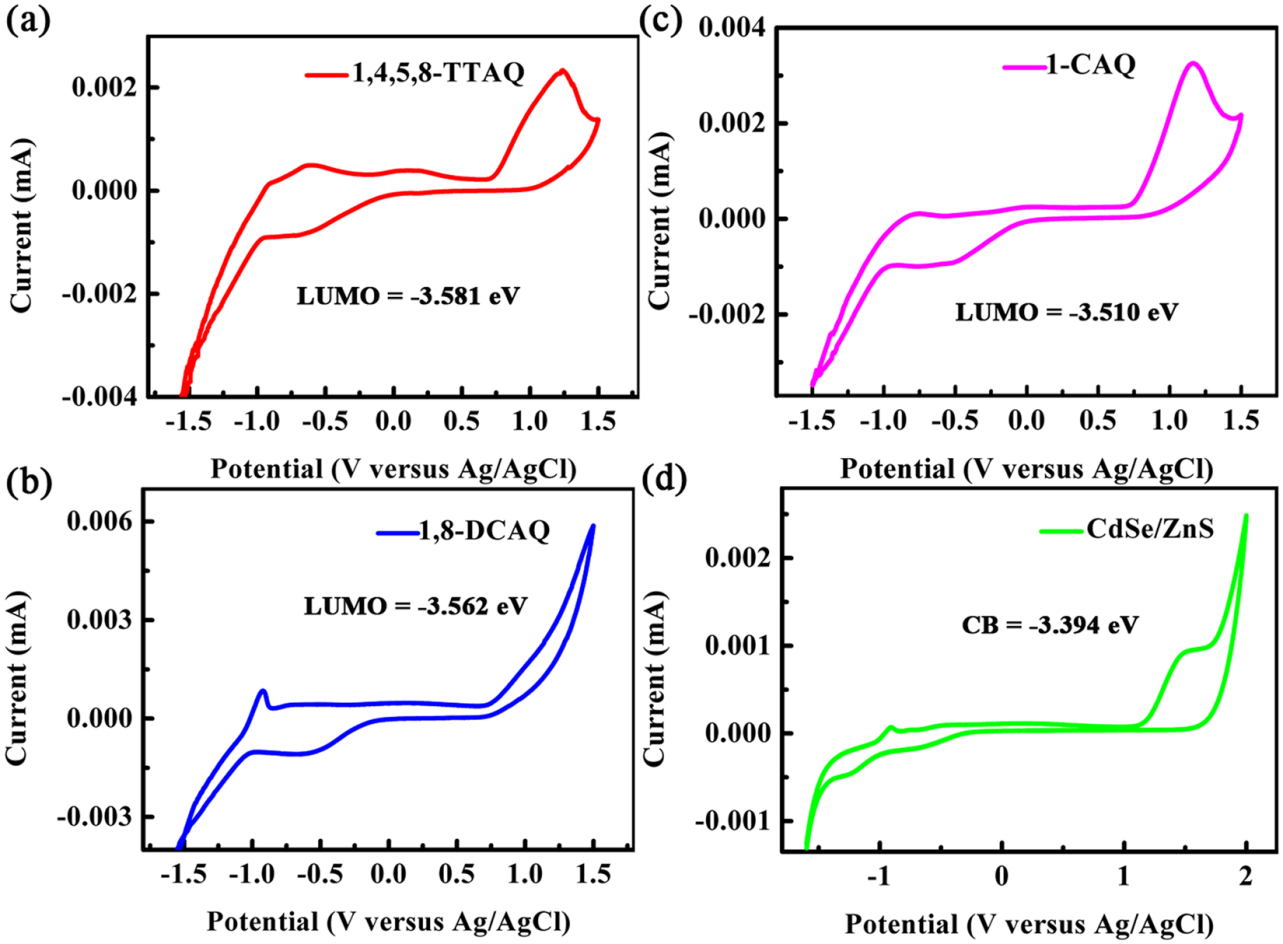
The cyclic voltammetry curves of 1,4,5,8-TTAQ (**a**), 1,8-DCAQ (**b**), 1-CAQ (**c**), and CdSe/ZnS QDs (**d**). The values of the LUMO and conduction band (CB) are listed in the caption.

**Figure 4:**
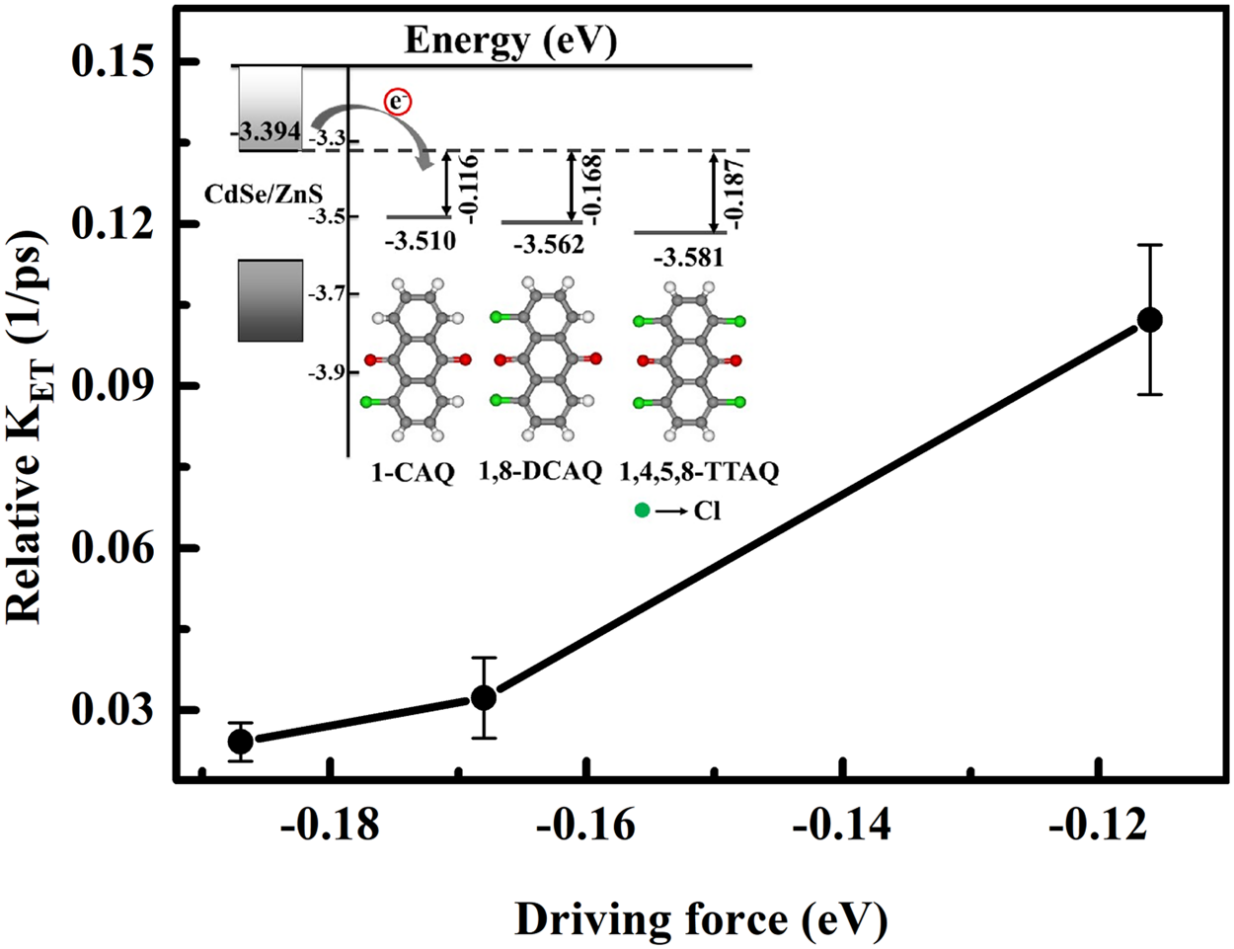
Dependence of the driving force on the decay rate. The inset is the schematic band diagram of CdSe/ZnS QDs and the LUMO potentials of different AQ derivative acceptor molecules.

The ΔG° depends on the highest occupied molecular orbital (HOMO) and lowest upper molecular orbital (LUMO) levels of the donor and acceptor components^33,34^. In our research systems, the ET ΔG° is related to the difference between the energies of the QD conduction band and the acceptor molecule LUMO^32^, resulting from the energy alignment between the QD conduction band edge and the LUMO of the AQ molecules. However, AQ derivatives act as good electron accepters to hold the electrons, so the LUMO of the AQ derivatives should be lower than that of the QDs^20^. The energy level alignment will result in an ET rate change. To confirm this, we carried out cyclic voltammetry (CV)^40–42^ measurements on the CdSe/ZnS QDs and AQ derivatives, as shown in Fig. 3. The conduction band value (−3.394 eV) of QDs and the LUMO values of 1,4,5,8-TTAQ (−3.581 eV), 1,8-DCAQ (−3.562 eV) and 1-CAQ (−3.510 eV) are shown in Fig. 3. The values measured in the experiments are in good agreement with the LUMO of the AQ derivatives theoretically calculated and shown in Table 3. The TA and PL results were combined to produce an energy level schematic diagram for the ET process and to illustrate the dependence of the energy shift (driving force) on the ET rate, as shown in Fig. 4, which provides further evidence for the above mechanism.

**Table 3:** The band energy LUMO potentials of different AQ derivative acceptor molecules.

System	LUMO potential (eV)
1,4,5,8-TTAQ	−3.243
1,8-DCAQ	−3.186
1-CAQ	−3.152

The energy band diagram of the ET process from the CdSe/ZnS QDs to the AQ derivatives is shown in the Fig. 4 inset. The conduction band energy of the CdSe/ZnS QDs is much higher than the LUMO potential of the three AQ derivatives, which ensures the ET process and the LUMO potential of the AQ derivatives increases as the number of chlorine substituents decreases. The energy level offset represents the ΔG^0^ for the ET from the CdSe/ZnS QD donor to the AQ derivative acceptors, i.e., the ΔG^0^ for QD-1,4,5,8-TTAQ, QD-1,8-DCAQ, and QD-1-CAQ systems is −0.187 eV, −0.168 eV, and −0.116 eV respectively. Based on these values, we drew Fig. 4 which reflects the dependence of the ET rate and driving force ΔG^0^. Figure 4 plots ΔG^0^ on the horizontal axis and the ET rate on the vertical axis. The ET rate is calculated by the expression k_ET_ = 1/τ, where τ is the ET lifetime^20,43^. The ET lifetime τ in the three systems can be obtained from a kinetic fit of the 1S_e_ bleaching signals and is shown in Fig. 2(d–f). From Fig. 4, we can clearly see that the ET rate increases as the driving force between the CdSe/ZnS QDs and AQ derivative acceptors increase. Specifically, the ET rate is also enhanced by a factor of 5 from 0.0241 to 0.1023 as the acceptor molecule changes from 1,4,5,8-TTAQ to 1-CAQ. That said, we modulate the LUMO potential of the acceptor molecule by changing the number of chlorine substituents, which is consistent with the fact that halogen atoms are frequently added to organic molecules to tune the LUMO potential because of their electronegativity^44^. Therefore, the driving force is the main reason for the change in the ET time caused by the addition of chlorine in our study, and acceptor molecules with good energy level matching should be selected to obtain an ET process with a much faster rate.

The last variable in Marcus Theory, electronic coupling H_DA_, is related to the distance between the donor and acceptor ((H_DA_)^2^∝exp(−βr_DA_))^45,46^ and the QD-molecule orbital shape^47^. For our three complexes, the H_DA_ values are similar because the distances between the QDs and AQ derivatives are almost the same because the radii of the AQ derivatives are nearly identical, as shown in Table 1. In addition, there is no change in the shape of the LUMO patterns of 1,4,5,8-TTAQ, 1,8-DCAQ, and 1-CAQ as indicated in Fig. S4. The consistent H_DA_ values are also similarly induced by the orbital shape of the three complexes. Combining the above studies on the distance between the QDs and AQ derivatives and the LUMO shapes of the AQ derivatives, we can conclude that there are few differences in the H_DA_ values of the three QD-AQ derivative systems and that the H_DA_ is not the main reason for the change in the ET time caused by the addition of chlorine in our study. In addition, it is directly difficult to calculate electronic coupling, i.e., H_DA_. We apply Eq. (1) and the known ET rate, RE, driving force to inversely infer the electron coupling in the three systems and the results are as shown in Table 4. The results indicate that H_DA_ is very small compared with the RE and driving force and that there is little difference in the H_DA_ values for the three systems. Our research can be used to develop a method to solve the difficulty of H_DA_ calculations.

**Table 4:** Calculation of electronic coupling H_DA_ for the three QD-AQ donor-accepter systems.

System	τ (ps)	λ (eV)	ΔG^0^ (eV)	H_DA_ (eV)
QDs-1,4,5,8-TTAQ	41.50	0.3938	−0.187	0.00162
QDs-1,8-DCAQ	30.79	0.4021	−0.168	0.00215
QDs-1-CAQ	9.78	0.4058	−0.116	0.00528

## Conclusion

In summary, we studied the photoinduced ET process in complexes based on CdSe/ZnS QDs and various AQ derivatives acting as electron acceptor molecules by femtosecond TA spectroscopy. The ET rate in QD-1,4,5,8-TTAQ, QD-1,8-DCAQ and QD-1-CAQ complexes can be enhanced by reducing the number of chlorine substituents. Consistent with Marcus ET theory, this enhancement is due to the increasing driving force, which is the energy level offset between the conduction band energy of the CdSe/ZnS QDs and the LUMO potential of the AQ derivatives, as confirmed by CV measurements. On the other hand, the electronic coupling between the QDs and AQ derivatives, and the total reorganization energy of the AQ derivatives and the solvent are not the main reasons for the change in the ET rate in the three systems. Our results provide a reference method to tune the ET process by selecting acceptors to design more efficient QD-based photovoltaic devices.

## Methods

CdSe/ZnS quantum dots were purchased from Suzhou Xinshuo Nanotech Co. (China). The acceptor molecules 1,4,5,8-TTAQ, 1,8-DCAQ, 1-CAQ, and CHX were purchased from Sigma (America), and J&K (China) and were used without further purification. The QD-AQ complexes were prepared by the addition of 1,4,5,8-TTAQ, 1,8-DCAQ, and 1-CAQ into CdSe/ZnS solutions in CHX based on methods detailed in a previous study^12^. Because AQ is insoluble in CHX, the solution was sonicated and filtered to ensure that all of the dissolved AQ was bound to the CdSe/ZnS QD surface. The concentration of QDs can be determined from the absorbance measured via steady-state absorption spectroscopy. The ratio of adsorbed 1,4,5,8-TTAQ, 1,8-DCAQ, and 1-CAQ to CdSe/ZnS can be considered to be approximately 3.5^12^, because the two research systems are very similar.

The steady-state absorption and fluorescence spectra of the QDs and QD-AQ complexes were measured using a 2550 UV-VIS spectrophotometer and RF5301 fluorescence spectrophotometer (Shimadzu), respectively. The femtosecond TA spectra and kinetics were recorded on a commercial system from Coherent (USA) and Ultrafast Systems (USA). The details of the ultraviolet and visible (UV-Vis) femtosecond transient absorption setup have been described elsewhere^48–50^. The femtosecond laser (Coherent Libra, USA) acts as a fundamental laser with a wavelength of 800 nm, a power of approximately 4 W at a 1 kHz repetition rate and the full width at half maximum of 50 fs is separated into two beams to generate pump and probe pulses at a 9:1 ratio. The excitation pump pulse is obtained to generate the second harmonic (λ_ex_ = 400 nm) of the fundamental laser via a 0.5 mm BBO crystal (β-BaB_2_O_4_, Fujian Castech Crystals Inc. China). The remaining beam is used to generate the white-light continuum via a sapphire plate (HELIOS, Ultrafast Systems, USA). The two beams are combined in a spectrometer (HELIOS) equipped with a computer-controlled delay line (up to 2 ns). Transient absorption kinetic traces were fitted using software from Ultrafast Systems (Surface Xplorer 2.2). The transient absorption experiments were conducted at room temperature.

All CV measurements were carried out on conductive glass sheets at a scan rate of 50 mV/s by a CHI 7100 electrochemical workstation. These sheets were covered with a uniform dispersion film of 1,4,5,8-TTAQ, 1,8-DCAQ and 1-CAQ and CdSe/ZnS QDs. The counter electrode was a platinum wire; the reference electrode was Ag/AgCl; the working electrode was glassy carbon in 0.5 mol/L Na_2_SO_4_ for 1,4,5,8-TTAQ, 1,8-DCAQ and 1-CAQ and Tetrabutylammonium perchlorate (TBAP) for the CdSe/ZnS QDs as the supporting electrolyte. The details have been described elsewhere^40,41,51–53^.

The reorganization, and volumes of 1,4,5,8-TTAQ, 1,8-DCAQ, and 1-CAQ were fully optimized using density functional theory (DFT)^54–56^. The B3-LYP (Becke’s three-parameter hybrid exchange function with Lee–Yang–Parr gradient-corrected correlation) function was used as the method and a 6–311 G basis was selected as the basis set^57–59^. In this study, all the electronic structure calculations were achieved using the Gaussian 09 program suite.

## Supplementary information

Supplementary information

## References

[1] Medintz IL, Uyeda HT, Goldman ER, Mattoussi H (2005). Quantum dot bioconjugates for imaging, labelling and sensing. Nat. Mater..

[2] Bruchez M, Moronne M, Gin P, Weiss S, Alivisatos AP (1998). Semiconductor nanocrystals as fluorescent biological labels. Science.

[3] Fan H (2005). Surfactant-assisted synthesis of water-soluble and biocompatible semiconductor quantum dot micelles. Nano Lett..

[4] Ocak I, Kara HES (2018). Phosphorescent detection of DNA-drug interaction based on emission quenching of ZnS quantum dots via photoinduced electron trasnfer. J. Lumin..

[5] Klimov VI (2006). Mechanisms for photogeneration and recombination of multiexcitons in semiconductor nanocrystals:  Implications for lasing and solar energy conversion. J. Phys. Chem. B.

[6] She C (2015). Red, yellow, green, and blue amplified spontaneous emission and lasing using colloidal CdSe nanoplatelets. ACS Nano.

[7] Kahane SV, Sudarsan V, Mahamuni S (2017). Anomalous photoluminescence enhancement due to hot electron transfer in core–shell Au–CdS nanocrystals. J. Lumin..

[8] Coe S, Woo W-K, Bawendi M, Bulović V (2002). Electroluminescence from single monolayers of nanocrystals in molecular organic devices. Nature.

[9] Enright MJ, Cossairt BM (2018). Synthesis of tailor-made colloidal semiconductor heterostructure. Chem. Commun..

[10] Kang B-H (2010). Highly efficient hybrid light-emitting device using complex of CdSe/ZnS quantum dots embedded in co-polymer as an active layer. Opt. Express.

[11] Pietryga JM (2004). Pushing the band gap envelope:  mid-infrared emitting colloidal PbSe quantum dots. J. Am. Chem. Soc..

[12] Zhu H, Song N, Lian T (2010). Controlling charge separation and recombination rates in CdSe/ZnS type I core−shell quantum dots by shell thicknesses. J. Am. Chem. Soc..

[13] Shao C (2013). Enhancement of electron transfer from CdSe core/shell quantum dots to TiO_2_ films by thermal annealing. J. Lumin..

[14] Ren S (2011). Inorganic–organic hybrid solar cell: bridging quantum dots to conjugated polymer nanowires. Nano Lett..

[15] Kirkeminde A, Scott R, Ren S (2012). All inorganic iron pyrite nano-heterojunction solar cells. Nanoscale.

[16] Huynh WU, Dittmer JJ, Alivisatos AP (2002). Hybrid nanorod-polymer solar cells. Science.

[17] Jhonsi MA, Thulasi S, Kathiravan A (2016). Impact of capping agent on the electron transfer dynamics of CdTe QDs with methyl viologen. J. Lumin..

[18] Diroll BT, Fedin I, Darancet P, Talapin DV, Schaller RD (2016). Surface-area-dependent electron transfer between isoenergetic 2D quantum wells and a molecular acceptor. J. Am. Chem. Soc..

[19] Masumoto Y, Takagi H, Umino H, Suzumura E (2012). Fast electron transfer from PbSe quantum dots to TiO_2_. Appl. Phys. Lett..

[20] Hyun B-R (2010). Role of solvent dielectric properties on charge transfer from PbS nanocrystals to molecules. Nano Lett..

[21] Zhu H, Lian T (2012). Wavefunction engineering in quantum confined semiconductor nanoheterostructures for efficient charge separation and solar energy conversion. Energy Environ. Sci..

[22] Zhu H, Song N, Rodríguez-Córdoba W, Lian WT (2012). Wave function engineering for efficient extraction of up to nineteen electrons from one CdSe/CdS quasi-type II quantum dot. J. Am. Chem. Soc..

[23] Olshansky JH, Ding TX, Lee YV, Leone SR, Alivisatos AP (2015). Hole transfer from photoexcited quantum dots: the relationship between driving force and aate. J. Am. Chem. Soc..

[24] Bi W (2015). Molecular co-catalyst accelerating hole transfer for enhanced photocatalytic H_2_ evolution. Nat. Commun.

[25] Masteri-Farahani M, Khademabbasi K (2018). Heavy atom quenching of CdS quantum dots photoluminescence: Evidences for electron transfer mechanism. J. Lumin..

[26] Huang J, Mulfort KL, Du P, Chen LX (2012). Photodriven charge separation dynamics in CdSe/ZnS core/shell quantum dot/cobaloxime hybrid for efficient hydrogen production. J. Am. Chem. Soc..

[27] Wu B (2015). Visible-light photoexcited electron dynamics of scandium endohedral metallofullerenes: the cage symmetry and substituent effects. J. Am. Chem. Soc..

[28] Wang H, Donegá C, Meijerink A, Glasbeek M (2006). Ultrafast exciton dynamics in CdSe quantum dots studied from bleaching recovery and fluorescence transients. J. Phys. Chem. B.

[29] Klimov VI, Mikhailovsky AA, McBranch DW, Leatherdale CA, Bawendi MG (2000). Quantization of multiparticle Auger rates in semiconductor quantum dots. Science.

[30] Klimov VI (2007). Spectral and dynamical properties of multiexcitons in semiconductor nanocrystals. Annu. Rev. Phys Chem..

[31] Gao Y, Sandeep CS, Schins JM, Houtepen AJ, Siebbeles LD (2013). Disorder strongly enhances Auger recombination in conductive quantum-dot solids. Nat. Commun.

[32] Zhu H (2014). Auger-assisted electron transfer from photoexcited semiconductor quantum dots. Nano Lett..

[33] Marcus RA (1964). Chemical and electrochemical electron-transfer theory. Annu. Rev. Phys. Chem..

[34] Marcus RA, Sutin N (1985). Electron transfers in chemistry and biology. Biochim. Biophys. Acta.

[35] Hirose T, Kutsuma Y, Kurita A, Kaneko T, Tamai N (2014). Blinking suppression of CdTe quantum dots on epitaxial graphene and the analysis with Marcus electron transfer. Appl. Phys. Lett..

[36] Marcus RA (1965). On the theory of chemiluminescent electron‐transfer reactions. J. Chem. Phys..

[37] Zou LY (2008). Theoretical study on photophysical properties of multifunctional electroluminescent molecules with different π-conjugated bridges. J. Phys. Chem. A.

[38] Li YY (2017). Screening and design of high-performance indoline-based dyes for DSSCs. RSC. Adv..

[39] Li Y, Xu B, Song P, Ma F, Sun M (2017). D–A−π–A system: light harvesting, charge transfer, and molecular designing. J. Phys. Chem. C.

[40] Inamdar SN, Ingole PP, Haram SK (2008). Determination of band structure parameters and the quasi-particle gap of CdSe quantum dots by cyclic voltammetry. ChemPhysChem.

[41] Amelia M, Lincheneau C, Silvi S, Credi A (2012). Electrochemical properties of CdSe and CdTe quantum dots. Chem. Soc. Rev..

[42] Haram SK, Quinn BM, Bard AJ (2001). Electrochemistry of CdS nanoparticles:  A correlation between optical and electrochemical band gaps. J. Am. Chem. Soc..

[43] Sharma SN, Pillai ZS, Kamat PV (2003). Photoinduced Charge Transfer between CdSe Quantum Dots and p-Phenylenediamine. J. Phys. Chem. B..

[44] Dou J-H (2015). Fine-tuning of crystal packing and charge transport properties of BDOPV derivatives through fluorine substitution. J. Am. Chem. Soc..

[45] Newton MD (2004). Bridge-mediated electron transfer and multiple reaction coordinates. Isr. J. Chem..

[46] Tarboush, N. A. Unraveling the mechanism and structural determinants of electron transfer through the Di-heme Enzyme MauG. 3515439 (2011).

[47] Kaledin AL, Lian T, Hill CL, Musaev DG (2015). A hybrid quantum mechanical approach: intimate details of electron transfer between type-I CdSe/ZnS quantum dots and an Anthraquinone molecule. J. Phys. Chem. B.

[48] Yin H (2016). A novel non-fluorescent excited state intramolecular proton transfer phenomenon induced by intramolecular hydrogen bonds: an experimental and theoretical investigation. Sci. Rep-Uk..

[49] Liu XC (2018). Pressure dependence of excited-state charge-carrier dynamics in organolead tribromide perovskites. Appl. Phys. Lett..

[50] Lin W (2017). Physical mechanism on exciton-plasmon coupling revealed by femtosecond pump-probe transient absoption spectroscopy. Materials Today Physics.

[51] Sun C, Li Y, Song P, Ma F (2016). An experimental and theoretical investigation of the electronic structures and photoelectrical properties of Ethyl red and Carminic acid for DSSC application. Materials.

[52] Dennany L (2011). Eletrochemiluminescence (ECL) sensing properties of water soluble core/shell CdSe/ZnS quantum dots/Nafion composite films. J. Mater. Chem..

[53] Shamsipur M (2007). Cyclic voltammetric, computational, and quantitative structure–electrochemistry relationship studies of the reduction of several 9,10-anthraquinone derivatives. Journal of Electroanalytical Chemistry.

[54] Zhao G-J, Han K-L (2007). Ultrafast hydrogen bond strengthening of the photoexcited fluorenone in alcohols for facilitating the fluorescence quenching. J. Phys. Chem. A.

[55] Zhou P (2012). The invalidity of the photo-induced electron transfer mechanism for fluorescein derivatives. Phys. Chem. Chem. Phys..

[56] Becke AD (1993). Density‐functional thermochemistry. III. The role of exact exchange. J. Chem. Phys..

[57] Frisch MJ, Pople JA, Binkley JS (1984). Self‐consistent molecular orbital methods 25. Supplementary functions for Gaussian basis sets. J. Chem. Phys..

[58] Andersson MP, Uvdal P (2005). New scale factors for harmonic vibrational frequencies using the B3LYP density functional method with the Triple-ζ Basis Set 6-311 +G(d,p). J. Phys. Chem. A.

[59] Montgomery JA, Frisch MJ, Ochterski JW, Petersson GA (1999). A complete basis set model chemistry. VI. Use of density functional geometries and frequencies. J. Chem. Phys.

