# Supplementary material for: Tunable electron transfer rate in a CdSe/ZnS-based complex with different anthraquinone chloride substitutes
Source: Sci Rep. 2019 May 23;9:7756. doi: 10.1038/s41598-019-44325-w (PMC6533304; doi:10.1038/s41598-019-44325-w)
Supplement: Supplementary file 1 — Supplementary information [file 41598_2019_44325_MOESM1_ESM.pdf]

Supplementary information

Tunable electron transfer rate in a CdSe/ZnS-based complex with different anthraquinone chloride substitutes

Huifang Zhao[1], Chaofan Sun[1], Hang Yin[1], Yuanzuo Li[*2], Jianbo Gao[*3], Ying Shi[*1] & Mengtao Sun[4]

[1]Institute of Atomic and Molecular Physics, Jilin University, Changchun 130012, China.

[2]College of Science, Northeast Forestry University, Harbin 150040, Heilongjiang, China.

[3] Ultrafast Photophysics of Quantum Devices Laboratory, Department of Physics and Astronomy, Clemson University, South Carolina, 29634, USA.

[4] School of Mathematics and Physics, Center for Green Innovation, Beijing Key Laboratory for Magneto-Photoelectrical Composite and Interface Science, University of Science and Technology Beijng, Beijing 100083, China.

*Corresponding authors: yzli@nefu.edu.cn (Y. ZL Li), jianbog@clemson.edu (J. Gao), shi_ying@jlu.edu.cn (Y. Shi).

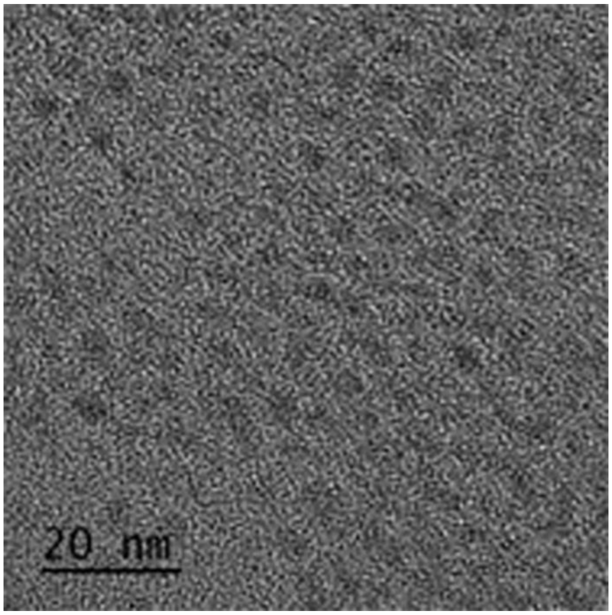

Fig. S1 | TEM (transmission electron microscope) image of the CdSe/ZnS used in our experiments with an approximately 5 nm in diameter (scale bar, 20 nm).

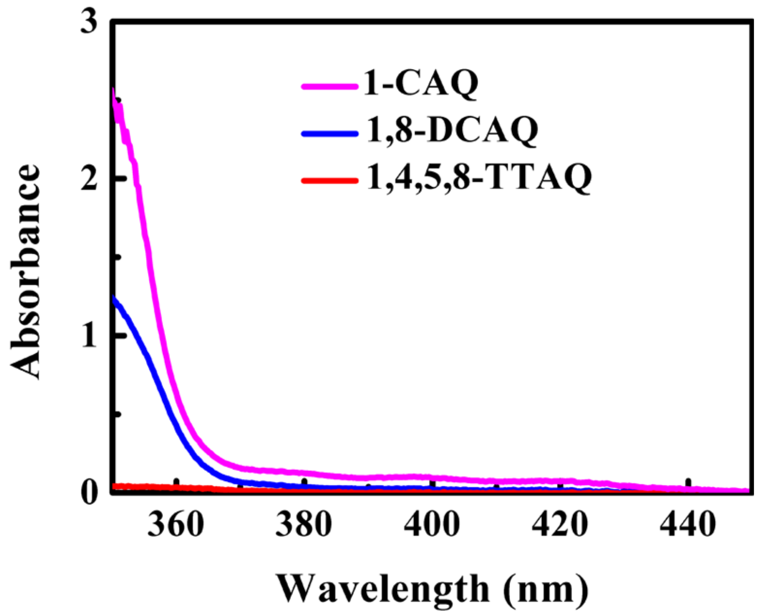

Fig. S2 | The steady-state absorption spectra of 1,4,5,8-TTAQ (red), 1,8-DCAQ (blue), 1-CAQ (pink) in cyclohexane (CHX).

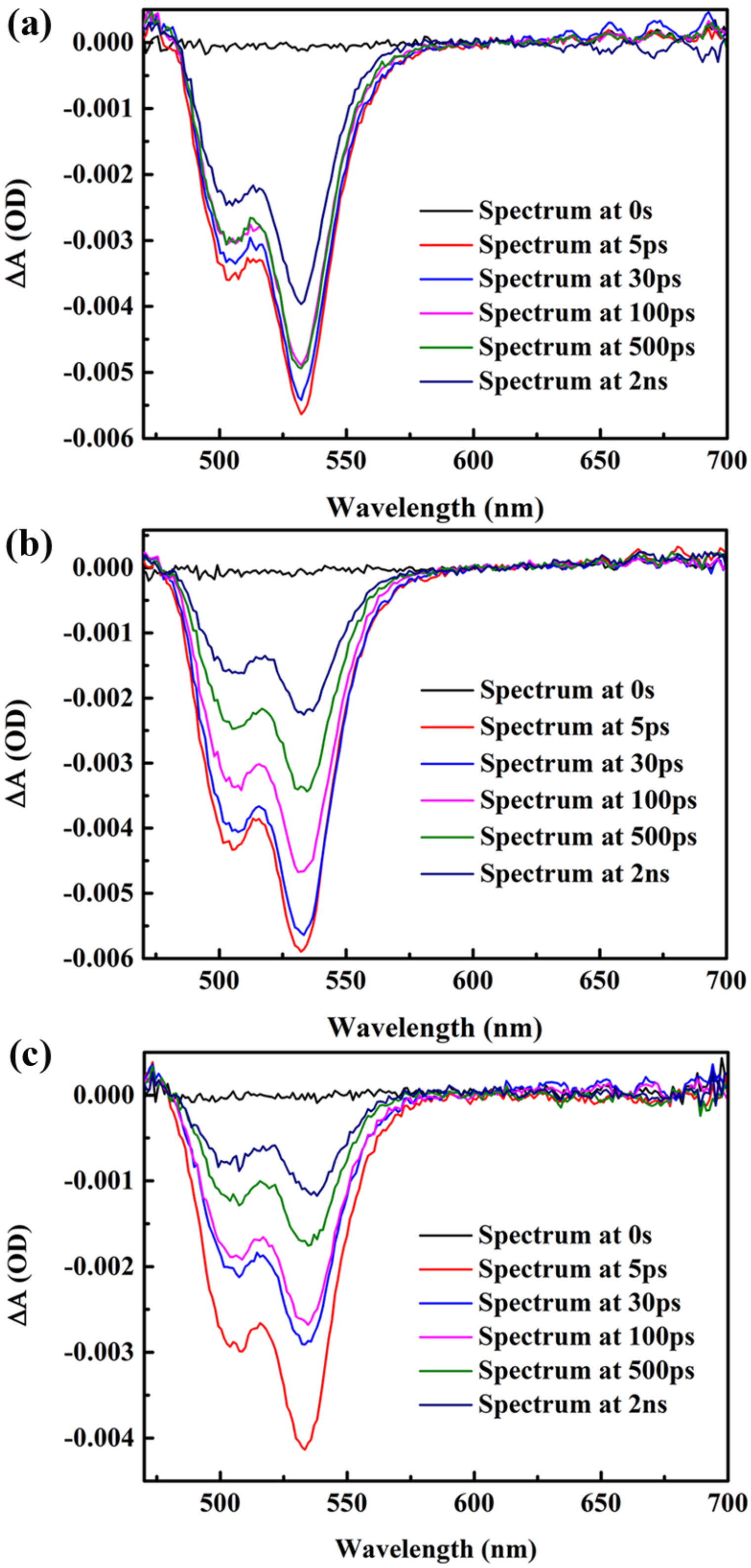

Fig. S3 | The transient absorption spectra of QD-AQ complexes at different delay times. QD-1,4,5,8-TTAQ (a), QD-1,8-DCAQ (b), QD-1-CAQ (c).

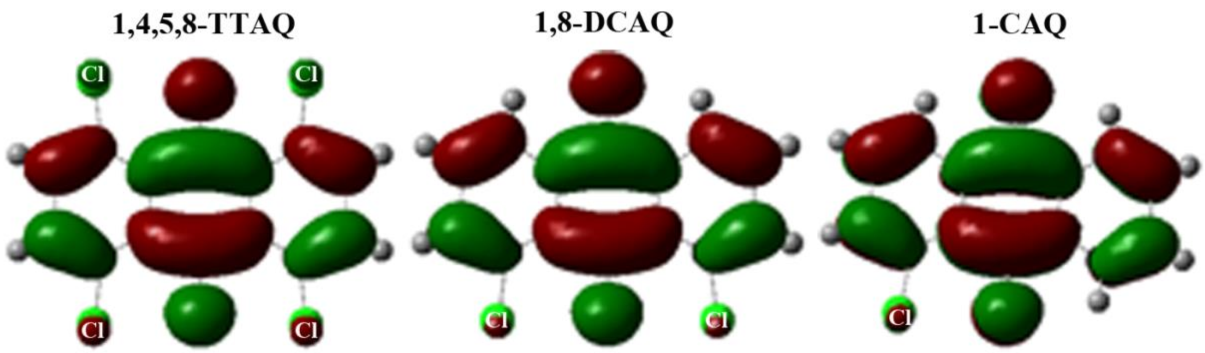

Fig. S4 | The LUMO patterns of 1,4,5,8-TTAQ, 1,8-DCAQ, and 1-CAQ。
